# Diagnostic performance of GeneXpert MTB/RIF in detecting MTB in smear-negative presumptive TB patients

**DOI:** 10.1186/s12879-022-07287-5

**Published:** 2022-04-01

**Authors:** Raksha Rimal, Dhiraj Shrestha, Susil Pyakurel, Rashmi Poudel, Prasha Shrestha, Kul Raj Rai, Gokarna Raj Ghimire, Ganesh Rai, Shiba Kumar Rai

**Affiliations:** 1 Department of Microbiology, Shi-Gan International College of Science and Technology, Kathmandu, Nepal; 2 Key Laboratory of Fujian-Taiwan Animal Pathogen Biology, College of Animal Sciences, Fujian Agriculture and Forestry University, Fuzhou, China; 3 National Tuberculosis Center, Bhaktapur, Nepal; 4 Department of Microbiology, Nepal Medical College and Teaching Hospital, Kathmandu, Nepal

**Keywords:** GeneXpert MTB/RIF, MTB, *Mycobacterium tuberculosis*, Nepal, Smear-negative

## Abstract

**Background:**

Tuberculosis (TB) is a leading cause of morbidity and mortality worldwide. Control of TB is lingering by the lack of diagnostic tests that are simple, rapid, yet accurate. Thus, smear-negative pulmonary TB often misses the diagnosis. The study evaluated the performance of GeneXpert MTB/RIF assay for the detection of *Mycobacterium tuberculosis* (MTB).

**Methods:**

The study was carried out from June to December 2016 in Nepal Tuberculosis Center, Bhaktapur, Nepal. A total of 173 sputum samples were collected and processed by microscopy [Auramine-O staining and Ziehl–Neelsen (ZN) staining], followed by GeneXpert MTB/RIF assay and culture in Lowenstein-Jensen (LJ) medium.

**Results:**

Of 173 sputum samples, 162 (93.6%) were smear-negative. Of 162 smear-negative sputum samples, 35 (21.6%) were confirmed to have MTB by culture, and 31 (19.1%) by GeneXpert MTB/RIF assay. Of 31 GeneXpert-positive samples, 25 (80.6%) were susceptible, 4 (12.9%) were resistant, and 2 (6.45%) were intermediate to rifampicin. The sensitivity, specificity, positive predictive value, and negative predictive value of GeneXpert MTB/RIF assay for smear-negative sputum samples were 74.3%, 96.6%, 86.7%, and 92%, respectively. The GeneXpert MTB/RIF has a substantial diagnostic agreement of 90.91% with culture (Cohen’s Kappa coefficient = 0.73).

**Conclusion:**

The diagnostic performance of GeneXpert MTB/RIF assay was almost on par with culture, and thus can be relied upon for MTB detection in smear-negative sputum samples.

## Background

Tuberculosis (TB), an infection caused by *Mycobacterium tuberculosis* (MTB), usually affects the lungs, but can also affect other parts of the body. Pulmonary TB (PTB) spreads easily by aerosols [1]. Globally, TB is a leading cause of death from a single infection. In 2019, there were an estimated 10 million new cases of TB, of which only 7 million cases were notified. Also, there were an estimated 1.4 million MTB attributed deaths. Similarly, there were an estimated 0.5 million new cases of rifampicin-resistant TB, of which 78% were multidrug-resistant TB (MDR-TB), but only 206,030 of MDR-TB were notified [2]. The lower efficacy of the diagnostic algorithm is one of the major reasons for lower notifications [2].

Sputum culture is the gold standard for the diagnosis of tuberculosis. However, it is expensive, contamination liable, labor-intensive, and time-consuming [3, 4]. This leads to delayed treatment and increased transmission [5]. Thus, early diagnosis plays a crucial role in the management and control of TB. Sputum smear microscopy is rapid and cheap, so is still used as the first-line technique for TB diagnosis, especially in resource-poor settings. But it lacks sensitivity (20–80%) and reproducibility [6, 7]. Therefore, employing an alternative rapid diagnostic tool is indispensable in the management of TB.

The GeneXpert MTB/RIF (Xpert) assay (Cepheid, Sunnyvale, CA, USA) is an automated nucleic acid amplification test. It can detect *M. tuberculosis* complex and rifampin resistance-associated mutation within two hours. In higher prevalence settings, Xpert has pooled sensitivity of 69.4- 84.7% and specificity of 98.4–98.8% [8, 9]; similarly, Xpert has pooled sensitivity of 60.6- 67% and a pooled specificity of 98.8% in smear-negative sputum samples [9, 10]. The technique is endorsed by the World Health Organization (WHO) as the point-of-care test for the diagnosis of both pulmonary and extra-pulmonary TB. However, full-fledged utilization of Xpert in low resource settings is challenging as it requires a constant electricity supply, a massive capital investment for devices and consumables, and persistent maintenance [11]. Nevertheless, with the subsidiary program from the global fund, Xpert testing is extensively being used in resource-limited settings with a higher TB burden. In Nepal, Xpert was first implemented in 2012 [12].

The poverty rate is high in Nepal [4], and poverty is a TB risk factor [6]. Almost half of the population in Nepal is presumed to have TB. In 2020, the total TB incidence was estimated to be 68,000, of which only 27,745 were notified, and TB attributed mortality was 17,220. This tantamount to 189 new cases, 101 missed cases, and 47 deaths every day [13]. The HIV-positive TB incidence in Nepal was 490, while HIV-positive TB mortality was 220 [13]. Since 2016, the nationwide ‘End TB Strategy’, has been implemented throughout the country. The treatment of TB is free of cost in Nepal. About NPR 1.8 billion (1 USD = NPR 118.5) were spent on TB in 2020 [13]. Still, the WHO recently enlisted Nepal in a group of 30 high MDR/RR- TB burden countries (MDR/RR-TB burden = 2,200) [13, 14]. Thus, on the premise of increasing TB prevalence in Nepal and the need for appropriate diagnostic algorithms, the study was conducted to assess the utility of Xpert for the rapid diagnosis of smear-negative TB in a high burden setting, Kathmandu in Nepal.

## Methods

### Study design, area, and sample population

The hospital-based cross-sectional study was carried out in the National Tuberculosis Center (NTC), Bhaktapur (located on the Eastern side of Kathmandu Valley), Nepal from June to December 2016. NTC is the focal point of the national TB program in Nepal. NTC formulate policies, strategies, planning, monitoring, and quality assurance of TB programs. The Central Laboratory at NTC is responsible for planning, training, monitoring, supervision, evaluation, and quality control of the laboratory network in the country. NTC also provides diagnostic services for all types of presumptive TB for the people of Kathmandu Valley and the rest of the country. Kathmandu valley is a heavily dense setting with the capital city, Kathmandu. The population of the valley is around 2.5 million, but the actual figure is around 4 million. The population density is 2,793. For this study, the sample population was presumptive TB cases of all age groups and genders visiting the center. The presumptive TB cases were patients with symptoms or signs suggestive of TB i.e. productive cough for two or more weeks accompanied by one or more of the following symptoms: night sweats, loss of appetite, fever, unintentional weight loss, chest pain, shortness of breath, fatigue, and malaise. Patients were included in the study if their sputum samples were smear-negative. Patients already under treatment for PTB, or had previously confirmed TB (1–3 years prior), and those suspected of extra-pulmonary tuberculosis were excluded from the study.

### Sample collection

A total of 173 sputum samples were collected from eligible patients. The patients were first counseled properly to produce a good sputum sample. Under supervision, two sputum samples were collected from each patient. For children and adults who were unable to expectorate sputum, sputum was induced using the nebulizer with hypertonic saline. The first specimen was collected on the spot and the second specimen was an early morning specimen of the next day (morning sample). The specimens were collected in a sterile, leak-proof, wide-mouthed, transparent plastic container (50 ml Falcon tube). The quality of the sputum sample was accessed macroscopically. The samples containing mostly saliva and nasal secretions were discarded and sample collection was repeated. The samples were well labeled and processed immediately or stored at 2 to 8ºC. The demographics of patients including age, sex, and smoking habits were also collected.

### Microscopy of the sputum samples

The sputum samples were observed microscopically for the presence of the Acid-Fast Bacilli (AFB) by auramine-O staining and Ziehl–Neelsen (ZN) staining [15]. If both specimens, i.e. spot and morning, from the same patient did not yield AFBs in both staining, then the sample was considered smear-negative. The smear-negative samples were further processed.

### Xpert MTB/RIF assay

All the procedures for the Xpert assay were conducted as per the manufacturer’s instructions [16]. In brief, the sample reagent (mixture of NaOH and isopropanol) in the ratio of 1:2 was added to sputum samples in the Falcon tube. The mixture was vortexed thoroughly until the clear solution was seen and incubated at room temperature for 15 min to minimize biohazard by reducing the viability of *M. tuberculosis*. Then, 2 ml of clear solution was added to the labeled cartridge with the help of a sterile dropper and the cartridge was incubated inside the Xpert machine. Results were obtained in the Xpert system within 2 h. The samples yielding positive results in at least 1 of the 2 cartridges of 2 sputum specimens, i.e. spot and morning, in Xpert was considered as the Xpert-confirmed TB.

### Culture of sputum

The sputum samples were digested and decontaminated by the modified Petroff method [17]. The digested sputum samples were inoculated on Lowenstein Jensen (LJ) media for culture. All the inoculated LJ media (slopes) were incubated at 37 °C for 8 weeks. The rough, buff, and tough growth of the LJ media was further subjected to ZN smear microscopy and biochemical tests to confirm *M. tuberculosis* [4]. *M. tuberculosis* was confirmed by their slow growth rate, colony morphology, inability to grow on LJ media containing *p*-nitrobenzoic acid (500 μg/ml), niacin test, and catalase test [4]. The samples yielding at least 1 of the 2 cultures of 2 sputum specimens, i.e. spot and morning, with *M. tuberculosis* growth was considered as the culture-confirmed TB. The culture-confirmed TB was considered as a definitive diagnosis.

### Statistical analysis

All the generated data were entered and curated by using Microsoft Excel version 2016. The statistical analyses were performed by using R software version 4.1.1. A descriptive analysis was used to describe the demographic variables. Descriptive statistics were expressed as percentages. The contingency of the categorical variables was observed by using the chi-square (χ^2^) test. Pearson’s Phi-coefficient was used to measure the effect size among the variables. Phi-coefficient was interpreted using Cohen's rules-of-thumb. A p-value < 0.01 was considered statistically significant. Both culture and smear-negative samples were considered as true TB negative. And, using culture-confirmed TB as the reference standard, the efficacy of the Xpert was assessed against the culture by calculating the estimate of measures i.e. sensitivity, specificity, positive predictive value (PPV), and negative predictive value (NPV). The estimates of measures were calculated using the formula as,

$${\text{Sensitivity}} = {\text{a}}/\left( {{\text{a}} + {\text{c}}} \right)$$

$${\text{Specificity}} = {\text{d}}/\left( {{\text{b}} + {\text{d}}} \right)$$

$${\text{PPV}} = {\text{a}}/\left( {{\text{a}} + {\text{b}}} \right)$$

${\text{NPV}} = {\text{d}}/\left( {{\text{c}} + {\text{d}}} \right)$ where, a = true positive, b = false positive, c = false negative and d = true negative.

The standard error (SE), the margin of error (M), and 95% Confidence Interval (CI) were calculated using the formula as,

$${95}\% {\text{ CI}} = {\text{p}} \pm { 1}.{96}*{\text{SE}}$$

$${\text{M}} = \left( {{\text{Upper CI}} - {\text{ Lower CI}}} \right)/{2}$$

${\text{SE}} = {\text{square root }}\left[ {\left\{ {{\text{p}}*\left( {{1} - {\text{ p}}} \right)} \right\}/{\text{N}}} \right]$ where p is an estimate of measures and N = number of true values of samples as per the gold standard.

All the estimates were expressed in percentages. Similarly, Cohen's Kappa coefficient (κ) was used to measure the inter-rater agreement of Xpert with culture reports. Cohen’s Kappa coefficient was interpreted using Cohen’s criteria. Cohen’s criteria used were the values ≤ 0 as indicating no agreement, 0.01–0.20 as none to a slight agreement, 0.21–0.40 as fair agreement, 0.41–0.60 as moderate agreement, 0.61–0.80 as substantial agreement, and 0.81–1.00 as almost perfect to perfect agreement.

## Results

Of 173 presumptive TB patients, 162 (93.6%) were smear-negative, while 11 (6.4%) were smear-positive. Of these 162 smear-negative presumptive TB patients, 35 (21.60%) were culture positive for MTB, while Xpert only detected MTB in 31 (19.14%) cases. Besides, 4 (2.47%) were confirmed to have rifampicin-resistant MTB (Table 1).

**Table 1:** Detection of MTB and rifampicin resistance by Xpert

Xpert reporting	Frequency N(%)
MTB NOT DETECTED	131 (80.86)
MTB DETECTED/Rif resistance NOT DETECTED	25 (15.43)
MTB DETECTED/Rif resistance INDETERMINATE	2 (1.23)
MTB DETECTED/Rif resistance DETECTED	4 (2.47)
Invalid test	9 (5.56)

MTB *Mycobacterium tuberculosis*, *Rif* Rifampicin, *TB* tuberculosis, *Xpert* GeneXpert MTB/RIF, the percentages were calculated on n = 162

The diagnostic performance of Xpert was almost similar to that of culture. There was no substantively significant association between Xpert assay and any of the demographics of patients (sex, age, smoking habits). Also, the same was the case for culture (Table 2).

**Table 2:** MTB detection by Xpert and culture as per demographics of patients

Demographics	Xpert-positive	χ^2^, *p*, φ	Culture-positive	χ^2^, *p*, φ
	N (%)		N (%)	
Sex				
Male (n = 114)	24 (21.05)	0.9136,	27 (23.68)	0.9821,
Female (n = 48)	7 (14.58)	0.339169*,	8 (16.67)	0.321679*,
		0.075		0.078
Age group				
≤ 20 years (n = 9)	3 (33.33)	3.4471,	3 (33.33)	1.7935,
21–40 years (n = 60)	14 (23.33)	0.327694*,	15 (25.00)	0.616351*,
41–60 years (n = 57)	10 (17.54)	0.146	10 (17.54)	0.105
≥ 61 years (n = 36)	4 (11.11)		7(19.44)	
Smoking habits				
Smokers (n = 99)	22 (22.22)	1.5672,	22 (22.22)	0.0573,
Non-smokers (n = 63)	9 (14.29)	0.210618*,	13 (20.63)	0.810862*,
		0.098		0.019
Total (n = 162)	31 (19.14)		35 (21.60)	

*MTB* *Mycobacterium tuberculosis*, *Xpert* GeneXpert MTB/RIF, *χ*^*2*^ chi-square statistic, *p* p-value, *φ* Phi-coefficient, the percentages were calculated taking n of the respective row as the divisor, *Denotes the test was not significant at p < 0.01

Of 162 smear-negative samples, 9 were false negative and 5 were false positive in Xpert. Taking culture as a reference, Xpert showed high diagnostic performance. Xpert showed Cohen’s Kappa coefficient (κ) of 0.73 (i.e. substantial agreement). This implies 90.91% agreement of Xpert with culture (Table 3) (Table 4).

**Table 3:** Diagnostic performance of Xpert taking culture as reference

Results	Culture-positive	Culture-negative	Total
Xpert-positive	26 (16.05%)	4 (2.47%)	30
Xpert-negative	9 (5.56%)	114 (70.37%)	123
Total*	35	118	153

*9 (5.56%) tests of 162 smear-negative sputum samples were invalid/error

**Table 4:** Diagnostic performance estimates of Xpert taking culture as reference

					95% CI	
Estimates	Values	N	SE	M	Upper	Lower
Sensitivity	74.29%	35	7.39%	14.48%	88.77%	59.81%
Specificity	96.61%	118	1.67%	3.27%	99.88%	93.34%
PPV	86.67%	30	6.21%	12.16%	98.83%	74.51%
NPV	92.68%	123	2.35%	4.60%	97.28%	88.08%
False positive rate	13.33%	30	6.21%	12.16%	25.49%	1.17%
False negative rate	7.32%	123	2.35%	4.60%	11.92%	2.72%
Cohen’s Kappa coefficient (κ): 0.73						

*Xpert* GeneXpert MTB/RIF, *SE* standard error, *M* margin of error, *95% CI* 95% Confidence Interval, *PPV* positive predictive value, *NPV* negative predictive value. The 9 invalid specimens were excluded from estimates calculation

## Discussion

TB is a global public health threat, especially in the third world. Thus, early detection is of utmost importance for reducing deaths and transmission. Global TB control is hampered by the lack of rapid and accurate diagnostic tests.

Smear microscopy is a rapid and cheap method to detect AFB, but at least 5,000–10,000 bacilli per mL of sputum should be present in sputum to yield a positive report in smear microscopy. The fewer bacilli will yield a negative report in smear microscopy. Thus, it is less sensitive. The 3-day early morning sputum specimens are required to increase sensitivity. The infectious dose of TB is lower than 10 bacilli, thus, smear microscopy can easily miss such cases. The smear-negative cases often miss diagnostic algorithms in low-resource settings, like Nepal. The smear-negative, culture-positive TB patients account for about 13% of TB transmission. Smear microscopy fails to differentiate MTB from MTB complex [18, 19]. Culture is still the gold standard for TB detection. It has high sensitivity and can detect MTB when 10 viable bacilli per mL of sputum are present. But, culture demands a longer time extending up to 4 weeks. Also, culture requires a biosafety level 3 laboratory [20, 21].

This premise makes Xpert a very reliable alternative. Xpert can improve diagnosis in smear-negative and some culture-negative TB. The mucopurulent sputum increases the Xpert yield [22]. The performance of Xpert was almost similar to culture in smear-negative presumptive TB cases, irrespective of the demographics of patients and smoking habits. The sensitivity, specificity, PPV, and NPV of Xpert in reference to the culture method were higher. The high diagnostic performance of Xpert has also been reported elsewhere [8, 9, 23]. Kim et al. [24] also reported similar findings. Similarly, Gowda et al. [25] also reported similar sensitivity, specificity, and NPV, except PPV. The specificity of Xpert was high, 96.61%. This can be influenced by the presence of false-positive cases, i.e. Xpert-positive culture-negative cases. Also, molecular techniques, like Xpert, can detect the DNA of MTB (both viable and non-viable), while only viable cells can show growth in culture. So, Xpert-positive does not necessarily imply viable bacilli. Thus, Xpert should not be used to monitor response to treatment, treatment failure, or relapse [22, 26, 27]. Besides, such cases may receive unnecessary treatment and receive delayed appropriate treatment [28]. The negative reports of one or more specimens in Xpert, smear microscopy [29], or even culture [30] do not necessarily exclude PTB. The high NPV of Xpert signifies that it can play a crucial role in decision-making to reduce airborne isolation of hospitalized presumptive TB cases. Besides, Xpert was also shown to be the most cost-effective option [31].

Xpert also detects rifampicin resistance. But, Xpert had shown the variable sensitivity and specificity to detect rifampicin resistance. The variation might be attributed to the false-positive rifampicin-resistant strains due to the genomic mutation, exclusion of mixed infections, and the occurrence of both rifampicin resistance and susceptible MTB isolates in the same samples [32]. Rifampicin resistance was very low in this study, thus limiting the true evaluation of Xpert to detect rifampicin resistance. Also, we could not confirm the rifampicin resistance detected by Xpert using drug susceptibility testing. This is the limitation of this study that rifampicin-resistant and susceptible cases by Xpert were not further confirmed.

Xpert has been in use in Nepal since 2012. Very few studies have been done in small clusters, still, there is a lack of comprehensive study of Xpert performance in pragmatic conditions in Nepal. It is now crucial to conduct the cost–benefit analysis in pragmatic settings if a single Xpert is enough; as a second Xpert only seems important in HIV infection. With the increasing expense, high rates of non-diagnostic results and no improvement in the proportion of patients starting treatment in high burden regions have been reported [33–35]. However, the higher running and installation cost of the Xpert needs to be holistically measured with the benefit it provides by avoiding the poor sensitivity and specificity of smear microscopy.

Nevertheless, the more rapid and accurate assay, Xpert, can aid physicians to make better and more informed decisions for the management of presumptive TB cases. The findings of the study strongly suggest Xpert is a valuable inclusion to the TB armamentarium, as a rapid assay, which can be combined with clinical findings for the initial PTB therapeutic decisions. This in turn can provide advances in TB control campaigns.

## Conclusions

The higher frequency of MTB in the smear-negative sputum sample signifies that authorities should keep eye on this domain for effective control of MTB. The diagnostic performance of the Xpert assay was almost on par with culture, and thus can be relied upon for MTB detection in smear-negative sputum samples.

## Limitations

First, the selection bias could have accounted for some errors in the findings. Second, the study was conducted for six months and the total samples were only 173. This is a comparatively small sample size. Thus, the generalization of the findings may not represent a real scenario. We believe that the research setting is the central TB laboratory of the country, so it represents the baseline scenario of the country as a whole. Even implying so, the larger sample population would give a better insight into the pragmatic diagnostic relevance of Xpert. Third, presumptive TB cases included in the study were not differentiated as per their severity. This could have affected the Xpert performance. Fourth, culture was used as the reference diagnostic test. Even though culture is still considered the gold standard, it too has limitations. Culture may also be falsely negative and if Xpert is positive, may not necessarily imply that the Xpert result is falsely-positive. Fifth, phenotypic drug susceptibility testing, line probe assay, and genotype MTBDRplus for all Xpert-positive specimens were not performed.

## Data Availability

The complete dataset generated and analyzed during the study is already covered in the text. The raw data can be made available at reasonable request to the corresponding author.

## Abbreviations

AFB: Acid-Fast Bacilli
LJ media: Lowenstein Jensen media
MDR-TB: Multidrug-resistant-tuberculosis
MDR/ RR-TB: Multidrug-resistant/ rifampicin-resistant tuberculosis
MTB: *Mycobacterium tuberculosis*
NTC: National Tuberculosis Center
PTB: Pulmonary tuberculosis
TB: Tuberculosis
WHO: World Health Organization
Xpert: Xpert MTB/RIF
ZN staining: Ziehl-Neelsen staining
